# Efficacy and Safety of Stem Cell Therapy for T1DM: An Updated Systematic Review and Meta-Analysis

**DOI:** 10.1155/2020/5740923

**Published:** 2020-10-10

**Authors:** Shi-Yi Sun, Yun Gao, Guan-Jian Liu, Yong-Kun Li, Wei Gao, Xing-Wu Ran

**Affiliations:** ^1^Diabetic Foot Care Center, Department of Endocrinology and Metabolism, West China Hospital Sichuan University, Chengdu, Sichuan 610041, China; ^2^Chinese Cochrane Centre, Chengdu, Sichuan 610041, China; ^3^Department of Liver Surgery and Liver Transplantation Center, West China Hospital Sichuan University, Chengdu, Sichuan 610041, China

## Abstract

**Background:**

The long-term insulin therapy for type 1 diabetes mellitus (T1DM) fails to achieve optimal glycemic control and avoid adverse events simultaneously. Stem cells have unique immunomodulatory capacities and have been considered as a promising interventional strategy for T1DM. Stem cell therapy in T1DM has been tried in many studies. However, the results were controversial. We thus performed a meta-analysis to update the efficacy and safety of stem cell therapy in patients with T1DM.

**Methods:**

We systematically searched the Medline, EMBASE, Cochrane Central Register of Controlled Trials, ClinicalTrials.gov, Web of Science, Wan Fang Data, China National Knowledge Infrastructure, VIP database, and the Chinese Biomedical Literature Database (SinoMed) for relevant studies published before March 19, 2019. The outcomes included parameters for glycemic control (i.e., glycosylated hemoglobin (HbA1c) levels and insulin dosages), *β* cell function (i.e., fasting C-peptide levels and area-under-curve of C-peptide concentration (AUCC)), and relative risk of adverse events. Statistical analysis was conducted by using RevMan 5.3 and Stata 12.0.

**Results:**

Five randomized controlled trials (RCTs) and eight nonrandomized concurrent control trials (NRCCTs) with a total of 396 individuals were finally included into the meta-analysis. Among RCTs, stem cell therapy could significantly reduce HbA1c levels (MD = −1.20, 95% CI -1.91 to -0.49, *P* = 0.0009) and increase fasting C-peptide levels (MD = 0.25, 95% CI 0.04 to 0.45, *P* = 0.02) and AUCC (SMD = 0.66, 95% CI 0.13 to 1.18, *P* = 0.01). Stem cell therapy could also reduce insulin dosages (SMD = −2.65, 95% CI -4.86 to -0.45, *P* = 0.02) at 6 months after treatment. NRCCTs also had consistent results. Furthermore, RCTs showed stem cell therapy did not increase relative risk of gastrointestinal symptom (RR = 0.69, 95% CI 0.14 to 3.28, *P* = 0.64) and infection (RR = 0.97, 95% CI 0.40 to 2.34, *P* = 0.95). However, NRCCTs showed stem cell therapy increased relative risk of gastrointestinal symptom (RR = 44.49, 95% CI 9.20 to 215.18, *P* < 0.00001).

**Conclusion:**

Stem cell therapy for T1DM may improve glycemic control and *β* cell function without increasing the risk of serious adverse events. Stem cell therapy may also have a short-term (3-6 months) effect on reducing insulin dosages.

## 1. Introduction

Type 1 diabetes mellitus (T1DM) occurs in children and adolescents mostly, which is acute-onset and prone to ketoacidosis whose typical symptoms are polydipsia, polyuria, and polyphagia with overt hyperglycemia. As one of the most common chronic childhood diseases in the world, the incidence of T1DM increases by about 3% annually globally [1]. T1DM is a chronic and immune-mediated disease, which is characterized by the permanent destruction of insulin-secreting *β* cells. [2]. Due to various acute and chronic complications, quality of life in patients with T1DM is severely decreased. And the life expectancy of patients with T1DM is evaluated to be decreased by about 12 years compared with the general population [3, 4]. Due to immune destruction of insulin-producing *β* cells, patients with T1DM have to rely on exogenous insulin to promote glucose utilization and storage and regulate glycogen breakdown. However, insulin may lead to hypoglycemia, obesity, and insulin resistance. Nowadays, pancreas or islet transplantation has gradually been used to treat T1DM, but autoimmunity, potential for surgical complications, and shortage of donor pancreas limit the development of this treatment [5].

Stem cells have unique immunomodulatory capacities and have been considered as a promising interventional strategy for various autoimmune diseases such as T1DM [6]. Yet the effect of stem cell therapy on glycemic control, insulin-secreting cells function, and exogenous insulin requirements in patients with T1DM remains a matter of controversy. Some studies showed that stem cell therapy added to insulin therapy reduced glycosylated hemoglobin (HbA1c) levels, and exogenous insulin dosages in patients with T1DM [7, 8]. Moreover, the use of stem cells had a positive effect on C-peptide secretion [7–9]. However, others indicated that stem cell therapy had no additional effect on HbA1c and *β* cell function in patients with T1DM [10]. In addition, the methodological defects including the combination of randomized controlled trials (RCTs) and single-arm trials or nonrandomized concurrent control trials (NRCCTs) existed in previous meta-analysis [11–13]. Therefore, we conducted a systematic review and meta-analysis to summarize the updated evidence on the efficacy and safety of stem cell therapy for T1DM in RCTs and NRCCTs, respectively.

## 2. Methods

Systematic review and meta-analysis was performed according to Preferred Reporting Items for Systematic Reviews and Meta-Analyses (PRISMA) [14](Table S5).

### 2.1. Literature Search and Data Extraction

Eligible studies were identified by consulting the Cochrane Library, PubMed, EMBASE, Web of Science, ClinicalTrials.gov, Wan Fang Data, CNKI, VIP, and SinoMed without date restriction and language restricted only in English and Chinese. Keywords and MeSH terms pertinent to the exposure of interest were used in relevant combinations: “diabetes Mellitus, Type 1,” “diabetes, autoimmune,” “diabetes mellitus, insulin-dependent,” “diabetes mellitus, sudden-onset,” “stem cells,” and “cell transplantation.” The literature search was run from inception to March 19, 2019 (Table S1).

RCTs and NRCCTs which were required to compare the effect of stem cell therapy versus placebo or no additional interventions to patients with T1DM were included in our analysis. Primary outcomes included HbA1c levels, insulin dosages, and the risk of adverse events after stem cell therapy. And the secondary outcomes were fasting C-peptide levels and area-under-curve of C-peptide concentration (AUCC). Studies were not included in the analysis if they met one of the following exclusion criteria: (1) case reports, reviews, animal and in vitro experiments, conference abstracts, and single-arm trials; (2) incomplete information about study objectives; and (3) studies in which data of outcomes were incomplete after contacting the corresponding author of the study in question. The PICOS (population, intervention, comparator, outcomes, and study design) approach was used to summarize the inclusion and exclusion criteria for qualitative/quantitative analyses. Two investigators screened titles and abstracts independently. Discrepancies were resolved through discussion or, if required, adjudication by a third author. Full texts of potentially relevant articles were then screened for inclusion in the final analysis after excluding nonrelevant studies. A standardized form was used to extract data from included studies for assessment of study quality and evidence synthesis. Extracted information included authors' name, year of publication, study design, number of patients, gender ratio, intervention, paths to treatment, mean dosages of stem cells, follow-up time, age, duration of T1DM, and study parameters. Outcomes' data were extracted as sample size and number of events for dichotomous variables or as mean and standard deviation (SD) for continuous ones.

### 2.2. Risk of Bias Assessment

Methodological quality of included RCTs was evaluated using the Cochrane Collaboration's tool [14]. For NRCCTs, the MINORS scale was used to assess the quality of studies meeting the inclusion criteria. The total score for the MINORS scale including twelve items is twenty-four stars as a maximum for the overall scale with the minimum of zero. Each item was scored from 0 to 2 (0 = not reported, 1 = inadequately reported, and 2 = adequately reported). A study was considered to include in meta-analysis if it achieved 13 out 24 and medium. Overall quality was independently determined by each reviewer with discrepancies solved by consensus [15].

### 2.3. Date Synthesis and Analysis

Statistical analyses were performed using Review Manager version 5.3, and Stata 12.0. Relative risk (RR) with 95% confidence intervals (CIs) was calculated to compare the risk of adverse events between the stem cell therapy group and the control group using the Mantel-Haenszel method. Mean difference (MD) with 95% CIs was used to compare fasting C-peptide levels and HbA1c levels. And standardized mean difference (SMD) with 95% CIs was used to compare AUCC and insulin dosages. Heterogeneity was evaluated by the Cochran's Q statistic and Higgins' and Thompson's *I*^2^ statistics. A fixed-effects or random-effects model was used to calculate the effect size depending on the heterogeneity results. The random-effects model was applied when heterogeneity existed (*I*^2^ > 50%); otherwise, the fixed-effects model was applied. Egger's regression test was performed to identify potential publication bias. We performed subgroup analyses according to the results at different follow-up times to explore the short-term and a relatively long-term efficiency of stem cell therapy.

## 3. Results

### 3.1. Literature Search and Study Selection

**Figure 1:**
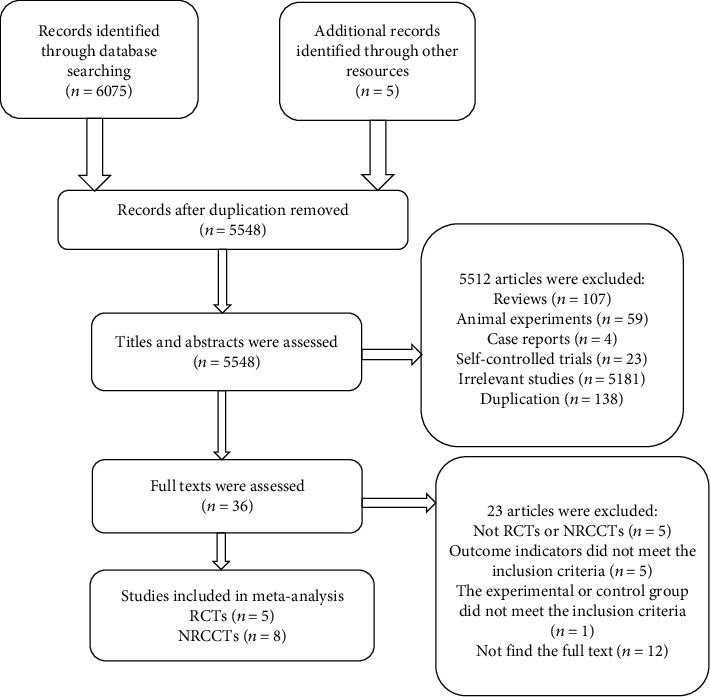
Flow chart of the selection of studies for the present systematic review and meta-analysis.

From the 6080 studies identified in our search, 532 duplicates were removed, and 5548 titles and abstracts were screened for eligibility. After full-text reviews, five RCTs and eight NRCCTs (including a total of 396 participants) were eligible for inclusion in the meta-analysis (Figure 1).

### 3.2. Study Characteristics

The characteristics of these studies are presented in Table 1. In RCTs, five studies were published from 2012 onwards. Patients' data were acquired from 154 participants with the age ranging from 17.6 to 27 years old. Only one study treated patients with hematopoietic stem cells [10], and four studies used mesenchymal stem cells [7, 9, 16, 17]. Moreover, only Cai et al. transplanted stem cells through the dorsal pancreatic artery or its substitute [8] while other research groups employed intravenous injection [7, 9, 10, 16]. The follow-up period ranged from 0.25 to 24 months, and the duration of T1DM ranged from 0.29 to 9.24 years.

In NRCCTs, a total of 242 patients with the age ranging from 8.04 to 33 years old were enrolled. Six NRCCTs [18–23] employed autologous hematopoietic stem cells to treat patients with T1DM while the other two used mesenchymal stem cells [17] and multipotent stem cells [24], respectively. In NRCCTs, all research groups employed intravenous injection to transplant stem cells. The follow-up period ranged from 1 to 48 months, and the duration of T1DM ranged from 0.14 to 8.5 years.

### 3.3. Risk of Bias Assessment

A summary of the risk of biases of included trials is reported in Tables 2 and 3. Random sequence generation is an important factor affecting the quality of studies. In the included RCTs, only one RCT was classified as unclear risk of bias because of unclear random sequence generation [7]. As shown in Table 3, most articles' total scores were 16, and other two articles' total scores were 14 and 18. Two NRCCTs [20, 24] were prospective collection of data, and two NRCCTs [20, 22] loss to follow-up more than 5%.

In order to assess the publication bias for the included studies, we chose outcomes including at least three studies to conduct the Egger's regression test. No publication bias was found in the outcomes we analyzed (Table S2 and Table S3).

### 3.4. Efficacy of Stem Cell Therapy for T1DM

#### 3.4.1. HbA1c Levels

**Figure 2:**
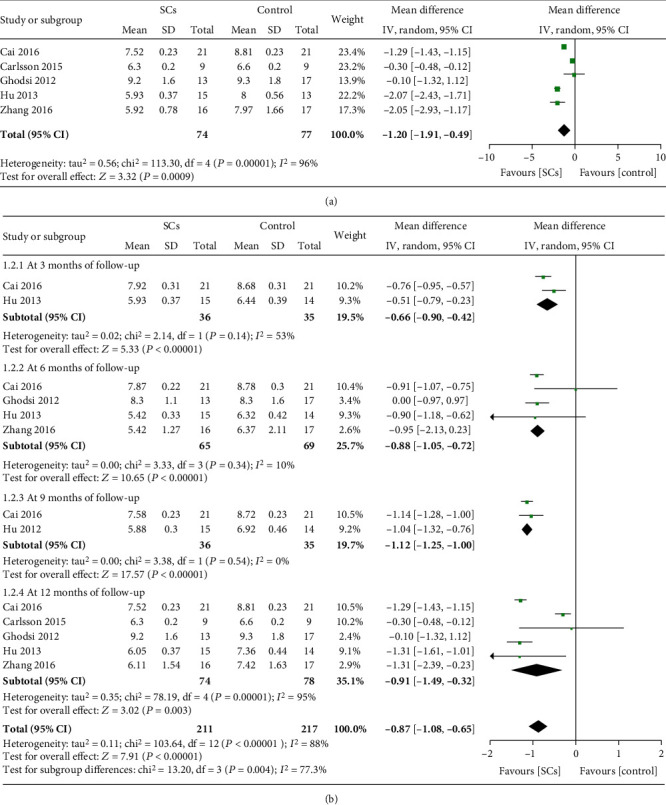
HbA1c levels at the longest follow-up (a). HbA1c levels at different follow-up (b) (RCTs).

All RCTs reported HbA1c levels after treatment. Statistical difference was found in RCTs demonstrating that stem cell therapy could decrease HbA1c levels compared with no additional treatment (MD = −1.20, 95% CI -1.91 to -0.49, *P* = 0.0009, *I*^2^ = 96%) (Figure 2(a)).

Patients who received stem cell therapy showed lower HbA1c levels than those receiving no additional treatment at 3, 6, 9, and 12 months in subgroup analysis (Figure 2(b)). Seven NRCCTs (*n* = 218) reported HbA1c levels [17–23]. However, HbA1c levels in the stem cell therapy group did not decrease compared with control group (MD = −0.42, 95% CI -1.09 to 0.26, *P* = 0.23, *I*^2^ = 74%) (Table S4).

#### 3.4.2. Insulin Dosages

**Figure 3:**
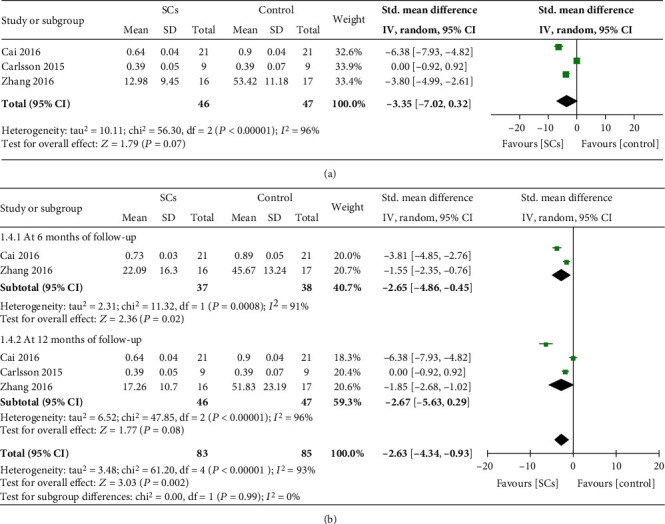
Insulin dosage at the longest follow-up (a). Insulin dosage at different follow-up (b) (RCTs).

Three trials [7, 8, 16] reported changes in insulin dosages. The statistical difference between the stem cell therapy group and the control group was not found (SMD = −3.35, 95% CI -7.02 to 0.32, *P* = 0.07, *I*^2^ = 96%) (Figure 3(a)). Of these trials, two RCTs (*n* = 75) reported insulin dosages at 6 months after treatment [7, 8], and all (*n* = 93) reported insulin dosages at 12 months after treatment [7, 8, 16]. Subgroup analysis show that, compared with controls, the stem cell therapy group had lower insulin dosages at 6 months (SMD = −2.65, 95% CI -4.86 to -0.45, *P* = 0.02, *I*^2^ = 91%) but had no lower insulin dosages at 12 months (SMD = −2.67, 95% CI -5.63 to 0.29, *P* = 0.08, *I*^2^ = 96%) (Figure 3(b)).

Five NRCCTs (*n* = 143) reported insulin dosages after treatment [17, 19, 20, 22, 23]. Insulin dosages in the stem cell therapy group decreased compared with controls (SMD = −0.36, 95% CI -2.35 to -0.37, *P* = 0.007, *I*^2^ = 83%) (Table S4).

#### 3.4.3. Fasting C-Peptide Levels

**Figure 4:**
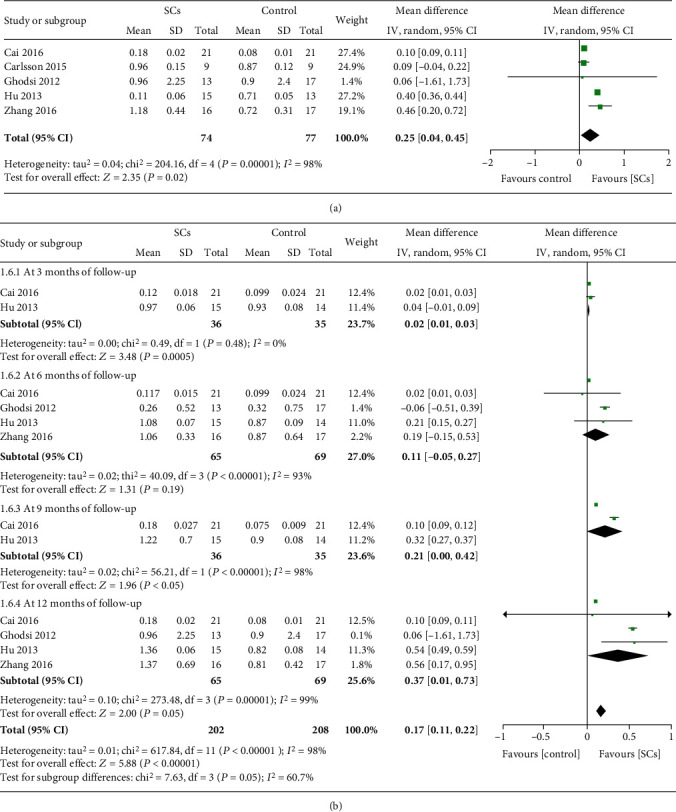
Fasting C-peptide at the longest follow-up (a). Fasting C-peptide at different follow-up (b) (RCTs).

Fasting C-peptide levels after treatment were reported in five RCTs [7–10, 16]. Compared with controls, fasting C-peptide levels in the stem cell therapy group significantly increased (MD = 0.25, 95% CI 0.04 to 0.45, *P* = 0.02, *I*^2^ = 98%) (Figure 4(a)). Subgroup analyses by different time points of follow-up showed that patients receiving stem cell therapy had higher fasting C-peptide levels than those receiving no additional treatment at 3, 9, and 12 months after treatment (all *P* ≤ 0.05) (Figure 4(b)). However, fasting C-peptide levels at 6 months were not significantly difference between patients receiving stem cell therapy and those receiving no additional treatment (MD = 0.11, 95% CI -0.05 to 0.27, *P* = 0.19, *I*^2^ = 93%) (Figure 4(b)). Four NRCCTs (*n* = 76) reported fasting C-peptide levels after treatment [17, 18, 23, 24]. The pooled results showed that fasting C-peptide levels in stem cell therapy group increased compared with controls (MD = 0.50, 95% CI 0.25 to 0.74, *P* < 0.0001, *I*^2^ = 69%) (Table S4).

#### 3.4.4. AUCC

**Figure 5:**
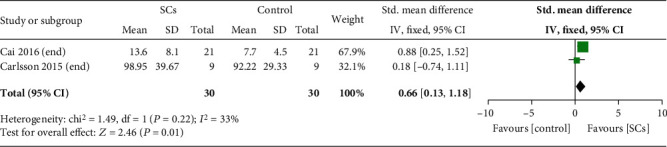
AUCC in experimental and control group (RCTs).

With regard to AUCC, two RCTs (*n* = 60) reported AUCC at terminal time (12 months) after treatment [8, 16]. AUCC in the stem cell therapy group increased compared with controls, and the heterogeneity was low (SMD = 0.66, 95% CI 0.13 to 1.18, *P* = 0.01, *I*^2^ = 33%) (Figure 5). Three NRCCTs (*n* = 102) reported AUCC [20, 21, 23]. A significant difference was observed between patients receiving stem cell therapy and those receiving no additional treatment, and the heterogeneity was high (SMD = 2.28, 95% CI 0.75 to 3.28, *P* = 0.004, *I*^2^ = 85%) (Table S4).

### 3.5. Risk of Adverse Events

The risk of adverse events was reported in five RCTs. Two RCTs showed that there were no adverse events after stem cell therapy [7, 10]. One study reported adverse events in the stem cell therapy group, including infection, bleeding, and abdominal pain [8]. Meta-analysis was performed according to the risk of infection and gastrointestinal symptom which were reported in three RCTs [8, 9, 16]. As shown in Table 4, there was no significant difference in the risk of infection and gastrointestinal symptom between the stem cell therapy group and the control group, respectively (RR = 0.97, 95% CI 0.40 to 2.34, *P* = 0.95, *I*^2^ = 45%; RR = 0.69, 95% CI 0.14 to 3.28, *P* = 0.64, *I*^2^ = 0%). Four NRCCTs reported adverse events after stem cell therapy, including febrile neutropenia, alopecia, blood component transfusions, autoimmune thyroid disease, irregular menstruation, gastrointestinal symptom, infection, primary hypothyroidism, fever, and thrombocytopenia [18–20, 22]. The most serious patient died of pseudomonas aeruginosa sepsis [18]. The risk of gastrointestinal symptom mentioned in three NRCCTs was pooled [19, 20, 22]. The results showed that compared with controls, the risk of gastrointestinal symptom in stem cell therapy group was higher (RR = 44.49, 95% CI 9.20 to 215.18, *P* < 0.00001, *I*^2^ = 0%) (Table 4).

## 4. Discussion

Our systematic review and meta-analysis found that, compared with placebo or no additional drugs, stem cell therapy could significantly decrease HbA1c levels at every time point of follow-up. After the 12 months' treatment, fasting C-peptide levels and AUCC were higher in the stem cell therapy group than the control group. This suggested that stem cell therapy could improve islet beta cell function through a relatively long-term treatment in patients with T1DM. However, the effect of stem cell therapy on reducing exogenous insulin dosages was not observed at 12 months in the meta-analysis. Thus, the transplantation route via the pancreatic artery rather than intravenous injection may be better choice for stem cell therapy in patients with T1DM.

Previous studies have revealed that stem cell therapy was more efficient at reducing HbA1c levels than insulin therapy alone in RCTs [8, 9]. In a meta-analysis including 9 RCTs and 13 self-controlled trials, stem cell therapy resulted in lower HbA1c levels than insulin therapy alone after 12 months treatment in RCTs or in self-controlled trials [12]. Similarly, in our systematic review and meta-analysis, the updated results from RCTs also showed that stem cell therapy exhibited effect on reducing HbA1c level either in the short-term (3-6 months) or in the relatively long-term treatment (9-12 months). Improved glycemic control could be attributed to rescuing *β* cell function or mass. As previously reported, among patients with T1DM, stem cell-based strategies represent significant therapeutic potential owing to the immunomodulatory potential and differentiation potentials of stem cells [6, 25]. These properties can potentially prevent *β* cell destruction, preserve residual *β* cell mass, and facilitate *β* cell regeneration [25–28].

In the present study, stem cell therapy significantly increased fasting C-peptide levels and AUCC compared with insulin therapy alone through the relatively long-term treatment (12 months). This suggests that the use of stem cells may improve islet *β* cell function or regeneration. Abnormal T cell-mediated immune response and the presence of chronic inflammatory infiltrate, which may result in the destruction of pancreatic islets, were considered as potential pathogenic mechanisms of the development of T1DM [29]. Evidence from in vitro studies showed that MSCs could correct Th1/Th2 imbalance and rebuild immune tolerance by upregulating the percentage of Treg cells and Th2 cells and downregulating the percentage of Th1 cells [30], MSCs transplantation could inhibit inflammatory response and maintain microenvironment homeostasis via regulating the phenotype of macrophages [31], and MSCs could differentiated into functional islet *β* cells by introducing 4 transcription factors, Pdx1, Ngn3, MafA, and Pax4 [32, 33]. Therefore, these mechanisms may be responsible for the increasing production and secretion of C-peptide. However, whether stem cell therapy could also improve islet *β* cell function in a short time (3-6 months) remains inconsistent. In the current meta-analysis, the pooled result of 2 RCTs at 3 months showed the increase of C-peptide secretion whereas the corresponding result of more RCTs at 6 months was on the opposite side. Thus, more evidences regarding the role of stem cell therapy in islet *β* cell function or regeneration during a short period of 3-6 months are warranted in future RCTs.

Interestingly, although C-peptide secretion (i.e., endogenous insulin secretion) increased as a result of stem cell therapy, the dosages of exogenous insulin did not significantly decrease in the stem cell therapy group than in the control group after 12 months of treatment. This may suggest that the increasing endogenous insulin secretion is not enough to reduce exogenous insulin dosages. However, the efficacy of stem cell therapy in improving glucose control, namely, the decrease of HbA1c, might be partly associated with the increasing endogenous insulin secretion. Of note, the pooled results of only two RCTs at 6 months showed that stem cell therapy could reduce exogenous insulin dosages. If only the two RCTs were considered at 12 months, stem cell therapy would also have similar results as that at 6 months. In fact, as more RCTs were included at 12 months, the decrease of exogenous insulin dosages in the stem cell therapy group was no longer observed. Thus, the inconsistent results between 6 months and 12 months may be attributed to fewer observational data from the included RCTs at 6 months. This reminds that both short-term follow-up points (3-6 months) and relatively long-term follow-up points (12 months) should be simultaneously set up in future RCTs so as to assess exact effect of stem cell therapy on exogenous insulin dosages over time.

Although safety in stem cell therapy has not been fully unraveled, there are increasing concerns relating to severe adverse effects such as the tumorigenic, embolization, severe hypoglycemia, and ketoacidosis [34–37]. In the current meta-analysis, however, the included RCTs did not observed the increasing risk of these severe adverse effects in the stem cell therapy group during the follow-up period. Considering some adverse events such as tumorigenic, it may need longer follow-up time to be observed; it is still necessary to further collect and evaluate the safety profile of stem cell therapy in father observation studies, particularly RCTs. In terms of other effects, NRCCTs indicated that stem cell therapy might cause more gastrointestinal discomforts, but the evidence from RCTs was not so. Due to limited numbers of included RCTs or reported cases, however, the evidence was weak. Despite this, those gastrointestinal discomforts may not cause significant harm to patients' health.

Of note, these RCTs included in the present study used diverse interventions, including different types of stem cells, cell number, route, and frequency of injection, which might have potential influence on the pooled results. In the included RCTs, most of the research group [7–9, 16] employed MSCs for the treatment of T1DM except for HSCs in only one RCT [10]. In a subgroup analysis, exclusion of the only one RCT (HSCs) did not change the observed effect of MSCs on glycemic control(HbA1c) and residual *β* cell function(fasting C-peptide), respectively (MD = −1.38, 95% CI -2.14 to -0.61, *P* = 0.0004, *I*^2^ = 97%; MD = 0.25, 95% CI 0.04 to 0.46, *P* = 0.02, *I*^2^ = 99%). Thus, among various types of stem cells, MSCs may be a feasible strategy to improve metabolic control and preserve *β* cell function in patients with T1DM. In terms of other stem cells, subgroup analysis was not performed due to limited number of included trials. In the included RCTs, most transplantation of stem cells was performed via intravenous route [7, 9, 10, 16] whereas only one RCT study used dorsal pancreatic artery rather than intravenous route to transplant stem cells [8]. When the only one RCT was removed, the effect of stem cell therapy on reducing HbA1c and increasing AUCC was no longer significant compared with controls, respectively (MD = −1.16, 95% CI -2.36 to 0.05, *P* = 0.06, *I*^2^ = 96%; SMD = 0.18, 95% CI -0.74 to 1.11, *P* = 0.70). This suggests that transplantation routes may also influence the therapeutic effect of stem cells. The data from in vivo studies showed that stem cell therapy by the pancreatic artery would be beneficial for homing of stem cells directly to the pancreas and avoiding from a pulmonary first-pass effect [38, 39], which may exert better therapeutic effect. Previous studies showed that the dose and injection frequency of stem cells may be associated with their therapeutic effect [40, 41]. In the present meta-analysis, patients in the included trials received different dose and injection frequency of stem cells, that is, different cell number. Due to the lack of consistence of cell number between these RCTs, however, we failed to evaluate the impact of the dose and injection frequency of stem cells on their therapeutic effect. More evidences regarding the role of the dose and injection frequency of stem cells on their therapeutic effect are warranted in future studies.

Our study has several strengths. In the present meta-analysis, the updated pooled results regarding the efficacy of stem cells therapy in patients with T1DM were mainly from data of RCTs which would contribute to produce more convincing evidences. We separately evaluated the efficacy of stem cell therapy in short-term (3-6 months) and a relatively long period (9-12 months) based on the different time points of follow-up, which was conductive to the observation of dynamic change of stem cell therapy. However, some limitations should be considered when interpreting our results. Firstly, some included trials lacked a rigorous approach and a complete reporting to ensure accuracy of the data. Secondly, among these RCTs included in our study, the sample size of study population, injection volume of stem cells, and duration of T1DM were significantly different, which may lead to high heterogeneity. However, limited cases and data hampered the conduction of subgroup analysis based on these differences. Thirdly, as only English and Chinese literatures were included in the meta-analysis, a potential publication bias may be introduced. Finally, due to limited number and limited information of the included studies, evidence of some important safety outcomes was weak. Considering rare adverse events or long-term adverse effects are unlikely to be observed in RCTs, the safety profile of stem cell therapy needs to be further collected and evaluated.

## 5. Conclusion

In conclusion, this systematic review and meta-analysis suggests stem cell therapy in patients with type 1 diabetes may reduce HbA1c and improve islet *β* cell function or regeneration. Although some NRCCTs but not RCTs indicated possible risk of increasing gastrointestinal discomforts, severe adverse effects caused by stem cells per se were not observed either in RCTs or NRCCTs. Therefore, stem cell therapy may offer a potential adjunct option to insulin monotherapy for T1DM. However, the current evidence for the abovementioned adverse effects is weak. More carefully designed and adequately powered RCTs are warranted to examine the effect of stem cell therapy on both short-term and long-term important outcomes.

## Figures and Tables

**Table 1:** Basic information of the included studies.

Author (year)	Country	Number (E/C)	Group	Female/male	Intervention	Treatment paths	Mean dose of stem cells (/kg)	Follow-up time (months)	Age (years)	Duration of T1DM (years)
Randomized controlled trials										
Cai, 2016 [8]	China	21/21	E^11^	12/9	aBM-MNCs^1^+UC-MSCs^2^+insulin	Via dorsal pancreatic artery or its substitute	aBM-MNCs (106.8 × 10^6^)+UC-MSCs (1.1 × 10^6^)	3, 6, 9, 12	NA^10^	9.24
			C^12^	10/11	Insulin	—	—		NA	7
Carlsson, 2015 [16]	Sweden	10/10	E	1/8	MSCs^3^+insulin	IV^9^	2.75 × 10^6^	2.5, 12	24 ± 2	NA
			C	4/5	Insulin	—	—		27 ± 2	NA
Ghodsi, 2012 [10]	Iran	13/17	E	6/7	HSCs^4^+insulin	IV	7 − 11 × 10^6^	0.25, 1, 3, 6, 12	21.61 ± 10.53	4.23 ± 2.21
			C	9/8	Insulin	—	—		21.35 ± 9.80	4.11 ± 2.86
Hu, 2013 [9]	China	15/14	E	6/9	WJ-MSCs^5^+insulin (twice, 4 weeks interval)	IV	2.6 ± 1.2 × 10^7^	1, 2, 3, 6, 9, 12, 15, 18, 21, 24	17.6 ± 8.7	NA
			C	6/8	Normal saline (twice, 4 weeks interval) + insulin	—	—		18.2 ± 7.9	NA
Zhang, 2016 [7]	China	16/17	E	7/9	ADMSCs^6^+insulin	IV	1 × 10^7^	6, 12, 24	22.1 ± 6.6	0.29 ± 0.24
			C	7/10	Insulin	—	—		21.6 ± 6.8	0.31 ± 0.56
Nonrandomized concurrent control trials										
Gu, 2018 [20]	China	20/20	E	7/13	AHSCT^7^+insulin	IV	NA	3, 6, 12, 18, 24, 36, 48	18 ± 3.9	0.19 ± 0.12
			C	7/13	Insulin	—	—		18 ± 4.5	0.14 ± 0.1
Gu, 2014 [19]	China	14/28	E	9/5	AHSCT+insulin	IV	NA	10.7 ± 4.2, 50.4 ± 21.6	8.04 ± 3.99	NA
			C	10/18	Insulin	—	—		8.29 ± 2.91	NA
Hou, 2014 [22]	China	15/25	E	7/8	AHSCT +insulin	IV	>3 × 10^6^	1.16 ± 0.1	18.95 ± 4.25	0.35 ± 0.09
			C	10/15	Insulin	—	—	1.18 ± 0.09	19.56 ± 4.62	0.38 ± 0.09
Walicka, 2018 [18]	Poland	23/8	E	—	AHSCT+insulin	IV	>3.0 × 10^6^	6, 12, 24, 36, 48	25 ± 5	NA
			C	—	Insulin	—	—		26 ± 3	NA
Wang, 2013 [21]	China	22/22	E	12/10	AHSCT+insulin	IV	NA	24	18.0 ± 4.2	NA
			C	12/10	Insulin	—	—		19.2 ± 3.5	NA
Ye, 2017 [23]	China	8/10	E	5/3	AHSCT+insulin	IV	NA	12	18.86 ± 1.46	NA
			C	6/4	Insulin	—	—		20.18 ± 4.02	NA
Yu,2011 [17]	China	6/6	E	3/3	UC-MSCs+insulin	IV	1 × 10^7^/person	9	19.67 ± 2.58	NA
			C	4/2	Insulin	—	—		14.83 ± 8.18	NA
Zhao, 2012 [24]	China	12/3	E	9/3	CB-SCs^8^+insulin	IV	NA	1, 3, 6, 10	28.17 ± 8.17	8.5 ± 5.42
			C	0/3	Insulin	—	—		33 ± 9	6 ± 7

aBM-MNCs: autologous bone marrow-derived mononuclear cells; UC-MSCs: umbilical cord-derived mesenchymal stem cells; MSCs: mesenchymal stem cells; HSCs: fetal liver-derived hematopoietic stem cells; WJ-MSCs: Wharton's jelly-derived mesenchymal stem cells; ADMSCs: allogeneic amniotic-derived mesenchymal stem cells; AHSCT: autologous hematopoietic stem cell transplantation; CB-SCs: human cord blood-derived multipotent stem cells; IV: intravenous; NA: not available; E: experimental group; C: control group.

**Table 2:** Methodological quality of the RCTs.

Author (year)	Random sequence generation (selection bias)	Allocation concealment (selection bias)	Blinding of patients and personnel (performance bias)	Blinding of outcome assessment (detection bias)	Incomplete outcome data (attrition bias)	Selective reporting (reporting bias)	Other bias
Cai, 2016	Low risk of bias	High risk of bias	High risk of bias	Low risk of bias	Low risk of bias	Low risk of bias	Unclear risk of bias
Carlsson, 2015	Low risk of bias	High risk of bias	High risk of bias	Low risk of bias	Low risk of bias	High risk of bias	Unclear risk of bias
Ghodsi, 2012	Low risk of bias	Unclear risk of bias	Low risk of bias	Low risk of bias	Unclear risk of bias	High risk of bias	Unclear risk of bias
Hu, 2013	Low risk of bias	Unclear risk of bias	Low risk of bias	Low risk of bias	Low risk of bias	High risk of bias	Unclear risk of bias
Zhang, 2016	Unclear risk of bias	High risk of bias	High risk of bias	Low risk of bias	Unclear risk of bias	Low risk of bias	Unclear risk of bias

**Table 3:** Methodological quality of the NRCCTs.

Author (year)	A	B	C	D	E	F	G	H	I	J	K	L	Total score
Gu, 2018	2	0	2	2	0	2	0	0	2	2	2	2	16
Gu, 2014	2	0	0	2	0	2	2	0	2	2	2	2	16
Hou，2014	2	0	0	2	0	2	0	0	2	2	2	2	14
Walicka, 2018	2	0	0	2	0	2	2	0	2	2	2	2	16
Wang, 2013	2	0	0	2	0	2	2	0	2	2	2	2	16
Ye, 2017	2	0	0	2	0	2	2	0	2	2	2	2	16
Yu,2011	2	0	0	2	0	2	2	0	2	2	2	2	16
Zhao, 2012	2	0	2	2	0	2	2	0	2	2	2	2	18

A: a clearly stated aim; B: inclusion of consecutive patients; C: prospective collection of data; D: endpoints appropriate to the aim of the study; E: unbiased assessment of the study endpoint; F: follow-up period appropriate to the aim of the study; G: loss to follow-up less than 5%; H: prospective calculation of the study size; I: an adequate control group; J: contemporary group; K: baseline equivalence of group; L: adequate statistical analyses.(0 = not reported, 1 = inadequately reported, and 2 = adequately reported).

**Table 4:** Incidence of adverse events in the experimental and control group (RCTs and NRCCTs).

Outcomes	No. of trials	Events/total		RR (95% CI)	*P* value	*I* ^2^
		Stem cells	Control			
Infection (RCTs)	3	7/45	7/43	0.97 (0.40, 2.34)	0.95	45%
Gastrointestinal symptom (RCTs)	3	1/45	2/43	0.69 (0.14, 3.28)	0.64	0%
Gastrointestinal symptom (NRCCTs)	3	10/44	0/73	44.49 (9.20, 215.18)	<0.00001	0%

## Data Availability

The data supporting this systematic review and meta-analysis are from previously reported studies and datasets, which have been cited.

## Supplementary Materials

Supplementary Materials Table S1: search strategy. Table S2: publication bias (RCTs). Table S3: publication bias (NRCCTs). Table S4: outcomes in experimental and control group (NRCCTs). Table S5: PRISMA checklist. (Supplementary Files).
