# Supplementary material for: Efficacy and Safety of Stem Cell Therapy for T1DM: An Updated Systematic Review and Meta-Analysis
Source: J Diabetes Res. 2020 Oct 10;2020:5740923. doi: 10.1155/2020/5740923 (PMC7569432; doi:10.1155/2020/5740923)
Supplement: Supplementary Materials — Table S1: search strategy. Table S2: publication bias (RCTs). Table S3: publication bias (NRCCTs). Table S4: outcomes in experimental and control group (NRCCTs). Table S5: PRISMA checklist. (Supplementary Files). [file 5740923.f1.docx]

|  | PubMed |
| --- | --- |
| #1 | (stem[Title/Abstract] OR hematopoietic[Title/Abstract] OR hemopoietin[Title/Abstract] OR progenitor[Title/Abstract] OR precursor[Title/Abstract] OR bone marrow[Title/Abstract] OR mononuclear[Title/Abstract] OR "adipose tissue"[Title/Abstract] OR mesenchymal[Title/Abstract] OR stromal[Title/Abstract] OR autologous[Title/Abstract] OR allogeneic[Title/Abstract] OR allogenic[Title/Abstract] OR ALDH*[Title/Abstract] OR C‐KIT*[Title/Abstract]) AND cell*[Title/Abstract] |
| #2 | cell transplantation[TA] OR stem cell*[TA] OR bone marrow transplant*[TA] |
| #3 | "autologous transplant*"[Title/Abstract] OR "cell therapy"[Title/Abstract] OR "cell therapies"[Title/Abstract] OR "cellular therapy"[Title/Abstract] |
| #4 | (cell[Title/Abstract] OR cells[Title/Abstract] OR cellular[Title/Abstract] OR myoblast*[Title/Abstract]) AND (transplant[Title/Abstract] OR transplantation[Title/Abstract] OR transplants[Title/Abstract] OR transplanting[Title/Abstract] OR transplanted[Title/Abstract] OR auto transplant*[Title/Abstract] OR allotransplant*[Title/Abstract] or graft*[Title/Abstract] OR implant[Title/Abstract] OR implants[Title/Abstract] OR implantation[Title/Abstract] OR implanted[Title/Abstract]) |
| #5 | "stem cells"[Mesh] |
| #6 | #1 OR #2 OR #3 OR #4 OR #5 |
| #7 | Diabetes mellitus, insulin-dependent[Title/Abstract] OR Diabetes mellitus, insulin dependent[Title/Abstract] OR Diabetes mellitus, juvenile onset[Title/Abstract] OR Diabetes mellitus, juvenile-onset[Title/Abstract] OR Diabetes mellitus, sudden onset[Title/Abstract] OR Diabetes mellitus, sudden-onset[Title/Abstract] OR Mellitus, sudden onset diabetes[Title/Abstract] OR Diabetes mellitus, type I[Title/Abstract] OR IDDM[Title/Abstract] OR Diabetes, juvenile onset[Title/Abstract] OR Diabetes, juvenile-onset[Title/Abstract] OR Diabetes mellitus, brittle[Title/Abstract] OR Diabetes mellitus, ketosis-prone[Title/Abstract] OR Diabetes mellitus, ketosis prone[Title/Abstract] OR Diabetes, autoimmune[Title/Abstract] OR "Diabetes Mellitus, Type 1"[Mesh] |
| #8 | #6 AND #7 |
| #9 | randomized controlled trial[Publication Type] OR controlled clinical trial[Publication Type] OR randomized[Title/Abstract] OR placebo[Title/Abstract] OR "drug therapy"[MeSH Subheading] OR trial[Title/Abstract] OR groups[Title/Abstract] OR randomly[Title/Abstract] |
| #10 | "Animals"[Mesh] NOT "Humans"[Mesh] |
| #11 | #9 NOT #10 |
| #12 | #8 AND #11 |

Table S1: Search strategy.

Table S2: Publication bias (RCTs).

| **Effect measure** | **HbA1c levels at terminal time** | **HbA1c levels at 6 months** | **Insulin dosages at terminal time** | **Insulin dosages at 12 months** | **Fasting C-peptide at terminal time** | **Fasting C-peptide at 6 months** | **Fasting C-peptide at 12 months** | **Incidence of gastrointestinal symptom** |
| --- | --- | --- | --- | --- | --- | --- | --- | --- |
| Statistical approach | Egger’s test | Egger’s test | Egger’s test | Egger’s test | Egger’s test | Egger’s test | Egger’s test | Egger’s test |
| P | 0.809 | 0.365 | 0.131 | 0.395 | 0.410 | 0.456 | 0.426 | 0.447 |

.

Table S3: Publication bias (NRCCTs)

| **Effect measure** | **HbA1c** | **Fasting C-peptide** | **Insulin dosages** | **AUCC** | **Incidence of gastrointestinal symptom** |
| --- | --- | --- | --- | --- | --- |
| Statistical approach | Egger’s test | Egger’s test | Egger’s test | Egger’s test | Egger’s test |
| P | 0.358 | 0.951 | 0.494 | 0.324 | 0.884 |

Table S4: Outcomes in experimental and control group (NRCCTs).

| **Number of**  **included studies** | **Effect measure** | **Total (95%)CI** | ***P* value** | **Heterogeneity I^2^** |
| --- | --- | --- | --- | --- |
| **HbA1c levels** | | | | |
| 7 | MD | -0.42[-1.09, 0.26] | 0.23 | 74% |
| **Insulin dosages** | | | | |
| 5 | SMD | -0.36[-2.35, -0.37] | 0.007 | 83% |
| **Fasting C-peptide levels** | | | | |
| 4 | MD | 0.50[0.25, 0.74] | <0.0001 | 69% |
| **AUCC** | | | | |
| 3 | SMD | 2.28[0.75, 3.28] | 0.004 | 85% |

Table S5: PRISMA checklist (This meta-analysis was followed the PRISMA statement, and PRISMA checklist was uploaded as supplementary materials.)

| **Section/Topic** | **#** | **Checklist Item** | **Reported on Page #** |
| --- | --- | --- | --- |
| **TITLE** |  |  |  |
| Title | 1 | Identify the report as a systematic review, meta-analysis, or both. | Page 1 |
| **ABSTRACT** |  |  |  |
| Structured summary |  | Provide a structured summary including, as applicable: background; objectives; data sources; study eligibility criteria, participants, and interventions; study appraisal and synthesis methods; results; limitations; conclusions and implications of key findings; systematic review registration number. | Page 1 to 2(line 14-38)  We did not report study appraisal, synthesis methods limitations and systematic review registration number. |
| **INTRODUCTION** |  |  |  |
| Rationale | 3 | Describe the rationale for the review in the context of what is already known. | Page 3 (line 40-66) |
| Objectives | 4 | Provide an explicit statement of questions being addressed with reference to participants, interventions, comparisons, outcomes, and study design (PICOS). | Page 3(line 64-66) |
| **METHODS** |  |  |  |
| Protocol and registration | 5 | Indicate if a review protocol exists, if and where it can be accessed (e.g., Web address), and, if available, provide registration information including registration number | Not report |
| Eligibility criteria | 6 | Specify study characteristics (e.g., PICOS, length of follow-up) and report characteristics (e.g., years considered, language, publication status) used as criteria for eligibility, giving rationale. | Page 3 to 4(line77-86) |
| Information sources | 7 | Describe all information sources (e.g., databases with dates of coverage, contact with study authors to identify additional studies) in the search and date last searched. | Page 4(line71-76) |
| Search | 8 | Present full electronic search strategy for at least one database, including any limits used, such that it could be repeated. | Supplementary Materials (Table S1) |
| Study selection | 9 | State the process for selecting studies (i.e., screening, eligibility, included in systematic review, and, if applicable, included in the meta-analysis). | Page 4 (line87-89) |
| Data collection process | 10 | Describe method of data extraction from reports (e.g., piloted forms, independently, in duplicate) and any processes for obtaining and confirming data from investigators. | Page 4(line89-95) |
| Data items | 11 | List and define all variables for which data were sought (e.g., PICOS, funding sources) and any assumptions and simplifications made. | Page 4(line78-81, 89-95) |
| Risk of bias in individual  studies | 12 | Describe methods used for assessing risk of bias of individual studies (including specification of whether this was done at the study or outcome level), and how this information is to be used in any data synthesis. | Page 4 to 5(line 97-102) |
| Summary measures | 13 | State the principal summary measures (e.g., risk ratio, difference in means). | Page 5(line 107-110) |
| Synthesis of results | 14 | Describe the methods of handling data and combining results of studies, if done, including measures of consistency (e.g., I2) for each meta-analysis. | Page 5(line 105-113) |
| Risk of bias across studies | 15 | Specify any assessment of risk of bias that may affect the cumulative evidence (e.g., publication bias, selective reporting within studies). | Page 5(line 113-114) |
| Additional analyses | 16 | Describe methods of additional analyses (e.g., sensitivity or subgroup analyses, meta-regression), if done, indicating which were pre-specified. | Page 5(line 114-116)  We added the methods of subgroup analyses to report our results better. |
| **RESULTS** |  |  |  |
| Study selection | 17 | Give numbers of studies screened, assessed for eligibility, and included in the review, with reasons for exclusions at each stage, ideally with a flow diagram. | Page 6(Fig. 1) |
| Study characteristics | 18 | For each study, present characteristics for which data were extracted (e.g., study size, PICOS, follow-up period) and provide the citations. | Page 8 to 11(Table 1 ) |
| Risk of bias within studies | 19 | Present data on risk of bias of each study and, if available, any outcome-level assessment (see Item 12). | Page 13 to 15(Table 2 and Table 3) |
| Results of individual  studies | 20 | For all outcomes considered (benefits or harms), present, for each study: (a) simple summary data for each intervention group and (b) effect estimates and confidence intervals, ideally with a forest plot. | Page 16 to 19(Fig. 2 & Fig. 3 & Fig. 4 & Fig. 5) |
| Synthesis of results | 21 | Present results of each meta-analysis done, including confidence intervals and measures of consistency. | Page 16 to 20(Fig. 2 & Fig. 3 & Fig. 4 & Fig. 5& Table 4) |
| Risk of bias across studies | 22 | Present results of any assessment of risk of bias across studies (see Item 15). | Supplementary Files (Table S2 and Table S3) |
| Additional analysis | 23 | Give results of additional analyses, if done (e.g., sensitivity or subgroup analyses, meta-regression [see Item 16]). | Page 16 to 19(Fig. 2 & Fig. 3 & Fig. 4 & Fig. 5) |
| **DISCUSSION** |  |  |  |
| Summary of evidence | 24 | Summarize the main findings including the strength of evidence for each main outcome; consider their relevance to key groups (e.g., health care providers, users, and policy makers). | Page 20 to 23(line 224-309) |
| Limitations | 25 | Discuss limitations at study and outcome level (e.g., risk of bias), and at review level (e.g., incomplete retrieval of identified research, reporting bias). | Page 23(line 316-325) |
| Conclusions | 26 | Provide a general interpretation of the results in the context of other evidence, and implications for future research. | Page23-24(line 306-310, 322-325) |
| **FUNDING** |  |  |  |
| Funding | 27 | Describe sources of funding for the systematic review and other support (e.g., supply of data); role of funders for the systematic review. | Page 24(line 340-345) |
